# A Threshold Equation for Action Potential Initiation

**DOI:** 10.1371/journal.pcbi.1000850

**Published:** 2010-07-08

**Authors:** Jonathan Platkiewicz, Romain Brette

**Affiliations:** 1 Laboratoire Psychologie de la Perception, CNRS and Université Paris Descartes, Paris, France; 2 Département d'Etudes Cognitives, Ecole Normale Supérieure, Paris, France; Université Paris Descartes, Centre National de la Recherche Scientifique, France

## Abstract

In central neurons, the threshold for spike initiation can depend on the stimulus and varies between cells and between recording sites in a given cell, but it is unclear what mechanisms underlie this variability. Properties of ionic channels are likely to play a role in threshold modulation. We examined in models the influence of Na channel activation, inactivation, slow voltage-gated channels and synaptic conductances on spike threshold. We propose a threshold equation which quantifies the contribution of all these mechanisms. It provides an instantaneous time-varying value of the threshold, which applies to neurons with fluctuating inputs. We deduce a differential equation for the threshold, similar to the equations of gating variables in the Hodgkin-Huxley formalism, which describes how the spike threshold varies with the membrane potential, depending on channel properties. We find that spike threshold depends logarithmically on Na channel density, and that Na channel inactivation and K channels can dynamically modulate it in an adaptive way: the threshold increases with membrane potential and after every action potential. Our equation was validated with simulations of a previously published multicompartemental model of spike initiation. Finally, we observed that threshold variability in models depends crucially on the shape of the Na activation function near spike initiation (about −55 mV), while its parameters are adjusted near half-activation voltage (about −30 mV), which might explain why many models exhibit little threshold variability, contrary to experimental observations. We conclude that ionic channels can account for large variations in spike threshold.

## Author Summary

Neurons communicate primarily with stereotypical electrical impulses, action potentials, which are fired when a threshold level of excitation is reached. This threshold varies between cells and over time as a function of previous stimulations, which has major functional implications on the integrative properties of neurons. Ionic channels are thought to play a central role in this modulation but the precise relationship between their properties and the threshold is unclear. We examined this relationship in biophysical models and derived a formula which quantifies the contribution of various mechanisms. The originality of our approach is that it provides an instantaneous time-varying value for the threshold, which applies to the highly fluctuating regimes characterizing neurons *in vivo*. In particular, two known ionic mechanisms were found to make the threshold adapt to the membrane potential, thus providing the cell with a form of gain control.

## Introduction

Spike initiation in neurons follows the *all-or-none* principle: a stereotypical action potential is produced and propagated when the neuron is sufficiently excited, while no spike is initiated below that threshold. The value of that threshold sets the firing rate and determines the way neurons compute, for example their coincidence detection properties [1], [2]. It is generally described as a voltage threshold: spikes are initiated when the neuron is depolarized above a critical value, when voltage-dependent sodium channels start to open. That biophysical mechanism is well understood since the studies of Hodgkin and Huxley in the squid giant axon [3] and subsequent modeling studies [4]–[7].

Recent findings have renewed the interest in the spike threshold. First, there is an intense ongoing debate about the origin of threshold variability observed in vivo [8]–[14]. In particular, it is unclear whether threshold variability is mainly due to experimental artifacts or molecular mechanisms, which might question the relevance of the Hodgkin-Huxley model for central neurons. Moreover, numerous experiments have shown that spike initiation does not only depend on the membrane potential but also on complex features of the inputs. For example, it depends on the preceding rate of depolarization [15]–[21] and on the preceding interspike intervals [12], [22]. Those properties are functionally important because they enhance the selectivity of neurons in several sensory modalities, in particular in audition [23], vision [24], and touch [21].

Developmental and learning studies have also shown that the threshold adapts to slow changes in input characteristics. This phenomenon is known as long-term plasticity of intrinsic excitability and may be involved in the regulation of cell firing, short term memory and learning [25]–[31]. The excitability threshold also varies with the distance to the soma in a given neuron and with cell type [2], [15], [32]–[35], which may explain functional differences.

The modulation of cell excitability might be explained by the activation of voltage-gated potassium channel Kv1 [36]–[41], inactivation of voltage-gated sodium channels [15], [16], [19], [21], fluctuations in sodium channel gating [42], inhibitory synaptic conductance [43]–[45] and the site of spike initiation [14]. To understand the origin of spike threshold variability, we examined the role of several candidate mechanisms in biophysical neuron models: activation and inactivation of the sodium channel, slow voltage-gated channels (e.g. Kv1), synaptic conductances and the site of spike initiation. Our analysis is based on a simplification of the membrane equation near spike initiation and results in a simple formula for the spike threshold that quantifies the contribution of all those mechanisms. The threshold formula provides an instantaneous time-varying value which was found to agree well with numerical simulations of Hodgkin-Huxley type models driven by fluctuating inputs mimicking synaptic activity *in vivo*, and with simulations of a realistic multicompartmental model of spike initiation [54].

## Results

### What is the spike threshold?

#### Spike threshold *in vitro*

**Figure 1:**
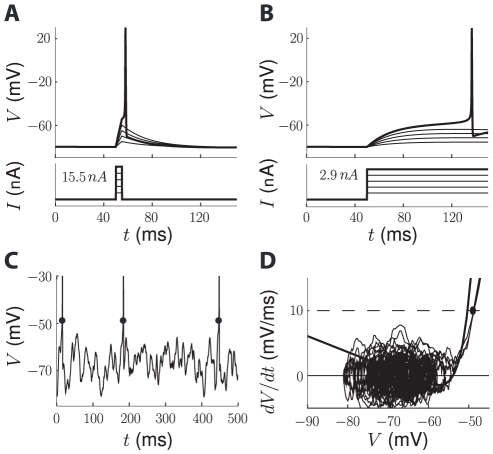
Spike threshold definitions. All plots were generated using the single-compartment model described in the Materials and Methods. A, *In vitro*, the neuron is stimulated with short current pulses with increasing intensity (bottom) and the threshold is the minimal value of that intensity above which the neuron spikes (top). The voltage threshold is the value of the membrane potential at that critical point. B, The threshold can be defined similarly with current steps (bottom) or other types of parameterized stimulations, yielding different values for the voltage threshold. C, *In vivo*, spike “threshold” is defined as a measure of the voltage at the onset of the action potential (black dots). The plot shows a simulated trace of a conductance-based model with fluctuating conductances (see Materials and Methods) and threshold is measured with the first derivative method. D, Representation of the trace in (C) in phase space, showing dV/dt vs. V. The first derivative method consists in measuring the membrane potential V when the derivative crosses a predefined value (dashed line) shortly before an action potential. The trace is superimposed on the excitability curve dV/dt = (F(V)+I_0_)/C, which defines the dynamics of the model. I_0_ is the mean input current, so that trajectories in phase space fluctuate around this excitability curve.

In a typical *in vitro* experiment, one measures the response of the cell to a controlled stimulus, whose strength is defined by a parameter (e.g. current intensity). The excitability threshold is then defined as the minimal value of this parameter above which a spike is elicited. Thus, the threshold is initially defined in stimulus space, for example as a charge threshold for short current pulses (Fig. 1A, simulated recording) or as a current threshold for current steps or ramps (Fig. 1B). The stimulus threshold corresponds to a voltage value, which we call the *voltage threshold*, but that value depends on the type of stimulation [46]. Nevertheless, we are interested in the voltage threshold rather than in the stimulus threshold because only the voltage is usually available in intracellular recordings *in vivo*.

#### Spike threshold *in vivo*

**Figure 2:**
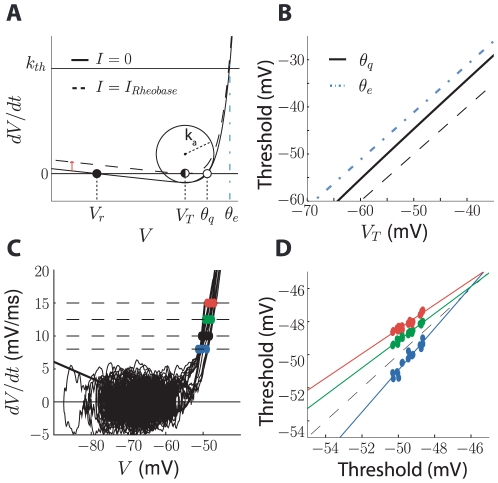
Relationships between spike threshold definitions. A, Excitability curve of the neuron model (dV/dt = (F(V)+I)/C; see Materials and Methods) for DC input current I = 0 (solid curve) and 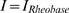 (dashed curve). With I = 0, the lower equilibrium (filled circle) corresponds to the resting potential V_r_, while the higher equilibrium (open circle) corresponds to the spike threshold with short pulses 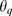 (as in Fig. 1A): if the membrane potential is quickly shifted above 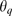, the membrane potential blows up and the neuron spikes (thus, this corresponds to the case when 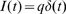, i.e., an impulse current). Slowly increasing the input current amounts to vertically shifting the excitability curve, and the membrane potential follows the resting equilibrium until it disappears, when 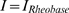. The voltage V_T_ at that point corresponds to the minimum of the excitability curve. The empirical threshold 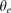 (with the first derivative method) is the voltage at the intersection of the excitability curve with the horizontal line dV/dt = k_th_ (dashed line). The slope threshold 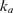 corresponds to the radius of curvature at V_T_. B, Threshold for short pulses 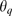 (solid line) and empirical threshold 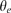 (blue dashed line) as a function of the threshold for slow inputs V_T_ (black dashed line is the identity line): the definitions are quantitatively different but highly correlated. C, Dependence of empirical threshold on derivative criterion k_th_: spike onsets are measured on a voltage trace (as in Fig. 1C) with derivative criterion k_th_ = 7.5 mV/ms (blue dots), 10 mV/ms (black), 12.5 mV/ms (green) and 15 mV/ms (red). D, Empirical threshold measured with k_th_ = 7.5 mV/ms (blue dots), 12.5 mV/ms (green) and 15 mV/ms (red) vs. threshold measured with 10 mV/ms, and linear regression lines. The dashed line represents the identity. The value of the derivative criterion (k_th_) impacts the threshold measure but not its relative variations.

Since the input to the neuron is not directly controlled in vivo, the concept of spike threshold does not have exactly the same meaning as in vitro. Rather, it is defined as the voltage at the “onset” of action potentials (Fig. 1 C), as observed on an intracellular recording of the membrane potential. Therefore the spike threshold is an empirical quantity that hopefully captures the same concept as *in vitro*, i.e., the point above which an action potential is initiated. Several measures of spike onset have been used in experimental studies [47]. The first derivative method consists in measuring the membrane potential V when its derivative dV/dt crosses an empirical criterion [8], [34] (Fig. 1D). The second and third derivative methods consist in measuring V when respectively d^2^V/dt^2^ and d^3^V/dt^3^ reach their maximum [12], [21]. Sekerli et al. (2004) compared those methods by asking electrophysiologists to identify spike onsets by eye on several membrane potential traces [47]. They found that visual inspection was best matched by the first derivative method, although that method critically relies on the choice of the derivative criterion (Fig. 2 C,D). However, all methods produced the same relative variations of the measured threshold.

#### Spike threshold in models

It might seem confusing that the definition of the voltage threshold is ambiguous and that most modulation effects that have been reported in the literature seem to apply to spike onset rather than spike threshold. However, as remarked in [47], those measures differ in absolute value but they vary in the same way. We can relate those definitions with a simple one-dimensional neuron model, where the membrane potential is governed by a differential equation:
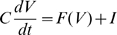
where C is the membrane capacitance, F(V) is the sum of all intrinsic voltage-dependent currents and I the input current. The dynamics of the membrane potential is determined by the excitability curve in phase space (dV/dt as a function of V, Fig. 2A). With no DC injected current (I = 0, solid curve), the differential equation has two fixed points, which are solutions of F(V) = 0: the lower one is stable and corresponds to the resting potential and the higher one is unstable and corresponds to the threshold for fast depolarizations (short current pulses, i.e., 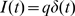), which we denote 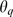. Indeed, after depolarization, the membrane potential V either goes back to the resting potential if 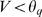 or keeps on increasing if 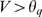, leading to a spike. If the neuron is progressively depolarized with a slowly increasing current, then the excitability curve slowly shifts upwards, depolarizing the stable potential, until the curve is entirely above zero and the neuron spikes (Fig. 2A, dashed curve). At that critical point, the curve is tangential to the horizontal axis and the voltage 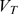 corresponds to the minimum of that curve: 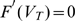. Thus, the voltage threshold for slow inputs (i.e., DC currents, or slow current ramps) is the solution of F′(V) = 0 and the voltage threshold for fast inputs (i.e., instantaneous charge inputs, or short current pulses) is the solution of F(V) = 0 with F′(V)>0.

The current-voltage function F(V) can be approximated by an exponential function near spike initiation (see Materials and Methods), leading to the exponential integrate-and-fire model [48]. In that model, we can calculate the relationship between the voltage threshold for slow inputs V_T_ and the voltage threshold for fast inputs 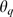 (see Text S1):
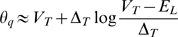
where 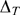 is the slope factor, characterizing the sharpness of spikes (see Materials and Methods). In single-compartment models, this is related to the slope of the Na activation curve. This formula provides a simple monotonous relationship between the two types of threshold, which is almost linear (the derivative of 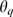 with respect to V_T_ is (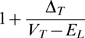), which is close to 1; see Fig. 2B). In our analysis, we chose the definition for slow depolarizations because it simplifies our formulas, but one can map the results to the definition for fast depolarizations using the formula above.

Empirical threshold measures used *in vivo* can be analyzed in the same way. For example, the voltage threshold measured by the first derivative method is the value 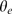 such that dV/dt = k_th_, i.e., the solution of 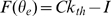. The empirical threshold can be approximately related to V_T_ with the following formula (see Text S1):
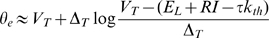
where 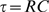 is the membrane time constant (R = 1/g_L_ is the membrane resistance). Although the relationship is more complex and shows a slight dependence on the input current I (thus increasing apparent threshold variability), it is still related with V_T_ through a monotonous (in fact quasi-linear) relationship and the choice of criterion k_th_ results mainly in a shift of the threshold, as shown in Fig. 2D.

In the remaining of this paper, we chose the voltage threshold for slow depolarizations V_T_ as the definition of the spike threshold (i.e., the voltage at current threshold).

### The threshold equation

#### Sodium channel activation

**Figure 3:**
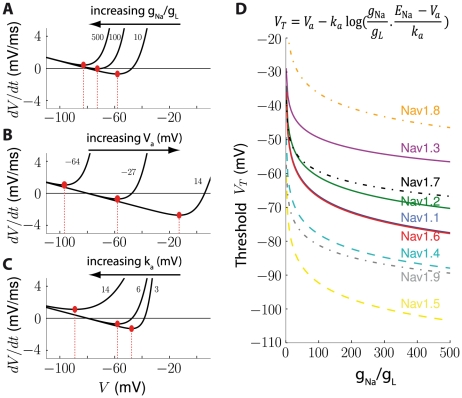
Influence of Na activation characteristics on spike threshold. A, Excitability curve of the model for different values of the ratio g_Na_/g_L_ (maximum Na conductance over leak conductance), discarding inactivation (h = 1) and other ionic conductances. The resulting threshold is shown with a red dot. B, Excitability curve for different values of half-activation voltage V_a_. C, Excitability curve for different values of Boltzmann factor k_a_. D, Threshold as a function of the ratio g_Na_/g_L_ for the 9 types of voltage-gated sodium channels [52] with characteristics reported in (Angelino and Brenner, 2007 [51]). For each channel type, the mean threshold obtained across the dataset is plotted. Nav1.[1], [2], [3], [6] are expressed in the central nervous system, Nav1.[4], [5] are expressed in cardiac and muscle cells and Nav1.[7], [8], [9] are expressed in the peripheral nervous system. Nav1.6 is expressed at the action potential initiation site [53]–[55].

Cells excitability is generally due to the presence of voltage-gated sodium channels [49]. More precisely, Na channel activation gates mediate a positive feedback mechanism, which produces the instability phenomenon necessary to initiate an action potential. Activation is very fast compared to all other relevant time constants (a fraction of ms), in particular the membrane time constant [50]. We make the approximation that it is instantaneous, so that the proportion of open sodium channels at any time is 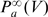. The membrane equation is then:
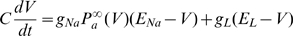
where g_Na_ (resp. g_L_) is the maximum Na conductance (resp. leak conductance) and E_Na_ (resp. E_L_) is the Na reversal potential (resp. leak reversal potential). We neglect inactivation and other ionic channels for the moment (see below). The activation function 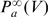 is well approximated by a Boltzmann function [51] with half-activation voltage V_a_ and activation slope factor k_a_. In the relevant part of that function, near spike initiation, it reduces to an exponential function and the membrane equation reads (see Materials and Methods):
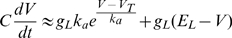
where
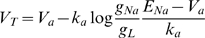
is the threshold (defined for slow inputs). The activation slope factor 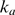 corresponds to the steepness of the Na activation curve, and characterizes the sharpness of spikes in single-compartment models (in the limit 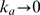 mV, the model tends to an integrate-and-fire model; it can be different in multicompartment models, see Discussion). The slope factor shows little variation across sodium channel types (k_a_ = 4–8 mV for neuronal channels, Angelino and Brenner, 2007 [51]). Thus, the threshold is primarily determined by the half-activation voltage and the density of sodium channels in log scale, relative to the leak conductance (see Fig. 3A–C).

This formula provides some quantitative insight about the role of Na channel on cell excitability. For example, Pratt and Aizenman (2007) observed that during development, tectal neurons adapt their intrinsic excitability to changes in visual input so as to stabilize output firing [28]. They hypothesized that this adaptation was mediated by regulation of Na channel density, which could be quantitatively evaluated using the formula above. Our formula also explains differences in excitability between cells. There are 9 Na channel types, which are expressed in different regions of the nervous system [52], and each one has specific properties, in particular specific values of V_a_ and k_a_. In Fig. 3D, we show how the threshold varies with channel density for each channel type, based on the dataset collected by Angelino and Brenner (2007) [51]. For the same channel density, the threshold can differ by up to 50 mV between channel types. Lowest threshold values were found for Nav1.5, expressed in cardiac cells, and highest ones were found for Nav1.8, expressed in dorsal root ganglion. Interestingly, among all channel types expressed in central neurons, the one with lowest threshold is Nav1.6, which is expressed in the spike initiation zone in the axon hillock [53]–[55].

#### Sodium channel inactivation and other conductances

The threshold can also be modulated by sodium channel inactivation and by the many other ion channels that can be found in neurons [56]–[58]. These factors might explain the effects of preceding spikes and membrane potential history on cell excitability [56]–[58]. To examine how they may modulate the threshold, we make two important assumptions: 1) inactivation is independent from activation, 2) these processes are slow compared to the timescale of spike initiation (about a millisecond). We then consider the membrane equation near spike initiation:
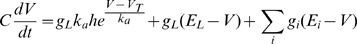
where h is the inactivation variable and g_i_ is the conductance of channel i, which may be voltage-gated (K+ channel) or synaptic. The contribution of additional ionic channels can be summed to yield an effective channel with conductance 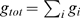 and reversal potential E* (see Materials and Methods), while the inactivation variable h can be entered into the exponential function:
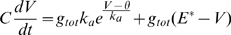
where
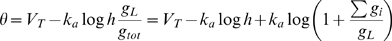
is the threshold (mathematically, it satisfies F′(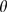) = 0, where F is the current-voltage function of the model). We call the formula above the *threshold equation*. It provides the instantaneous value of the spike threshold as a function of the sodium inactivation variable h (1-h is the proportion of inactivated Na channels) and of the other ionic channel conductances, including synaptic conductances. To obtain this equation, we made a quasi-static approximation, i.e., we assume that all modulating variables (h and g_i_) vary slowly at the timescale of spike initiation. We note that the threshold is determined by the value of conductances relative to the leak conductance rather than by their absolute value.

**Figure 4:**
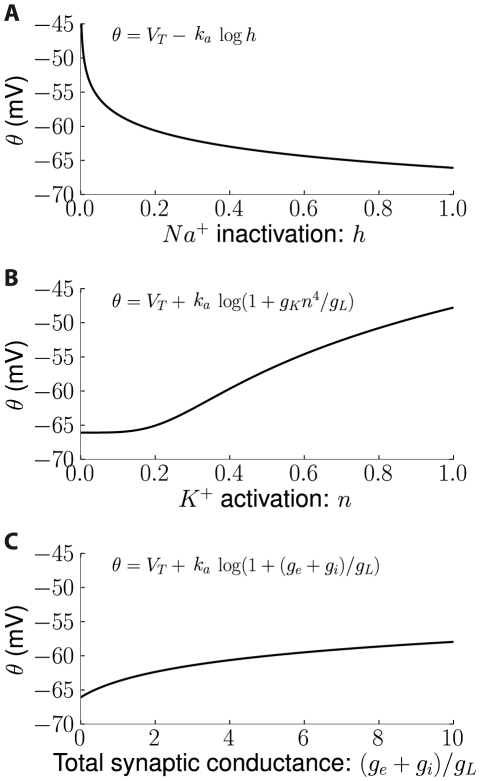
Influence of Na inactivation and ionic conductances on spike threshold in the conductance-based model (k_a_ = 3.4 mV, see Materials and Methods). A, Spike threshold θ as a function of Na+ inactivation variable h, with all other ionic conductances suppressed. B, Threshold as a function of K+ activation variable n, without inactivation (h = 1). C, Threshold as a function of total synaptic conductance (excitatory g_e_ and inhibitory g_i_), relative to the resting conductance g_L_ (conductances are considered static).

Fig. 4 illustrates the dependence of threshold on Na inactivation and conductances. As expected, the threshold increases when h decreases, that is, when more Na channels inactivate. It also increases with the total non-sodium conductance, which is also intuitive: more Na conductance is required to produce a spike when the other conductances are larger. Threshold modulation is proportional to the slope factor 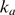, which shows little variation across Na channel types (4–8 mV in neuronal channels).

The threshold equation predicts several effects. Spike threshold should be higher *in vivo* than *in vitro* because the total conductance is several times larger [59]. For the same reason, it should also be higher in up states than in down states. It is correlated with sodium inactivation, so that it should increase with the membrane potential, as observed *in vitro* and *in vivo* [15], [16], [54]. Besides, threshold modulation by inactivation is strongest when many Na channels are inactivated (h close to 0), that is, when the neuron is depolarized. Spike threshold is correlated with voltage-gated conductances such as those of K+ channels. For high-threshold K+ channels with large conductance, the spike threshold increases by 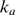 when the membrane potential increases by k_a_^K+^ (slope of K+ channel activation function). Indeed, far from half-activation value V_a_^K+^, the K+ activation curve is approximately 
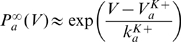
, which implies that threshold modulation is 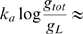
constant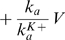 (provided K+ conductance is large enough). It also increases after each action potential (see below). Inactivation and adaptive voltage-gated conductances (e.g. Kv1) have similar effects but inactivation is “invisible”, in the sense that it affects excitability without changing the membrane potential or the total conductance.

### Threshold dynamics

To derive the threshold equation, we made a quasi-static approximation, assuming that all mechanisms that modulate the threshold are slow processes (compared to the timescale of spike initiation). That threshold equation provides an instantaneous value of the spike threshold, as a function of modulating variables. Here we show how the dynamics of sodium inactivation, voltage-gated conductances and synaptic conductances translate into spike threshold dynamics.

#### Sodium inactivation

Several authors have hypothesized that Na inactivation is responsible for experimentally observed threshold variability *in vivo* [12], [15], [16], [21]. We have shown that the instantaneous value of the spike threshold depends on the value of the inactivation variable h (1-h is the proportion of inactivated channels). We assume, as in the Hodgkin-Huxley model, that h evolves according to a first-order kinetic equation:
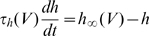
where 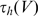 is the time constant and 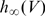 is the equilibrium value. This differential equation translates into a differential equation for the threshold θ (see Materials and Methods), which can be approximated by a similar first-order kinetic equation :
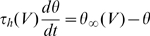
where 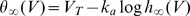 is the equilibrium value of the threshold. A linearized version of this equation was recently proposed as a simplified model of post-inhibitory facilitation in brainstem auditory neurons [60]. This is also consistent with previous results *in vitro* showing that the instantaneous value of the threshold increases with the membrane potential [61].

**Figure 5:**
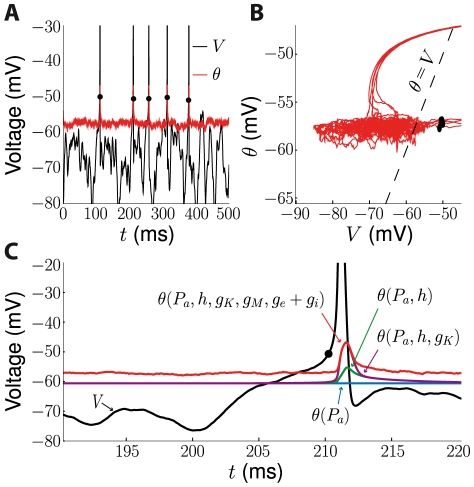
Dynamical spike threshold. A, Voltage trace of the fluctuating conductance-based model (black line) and predicted threshold according to our threshold equation (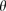, red line), calculated continuously as function of h, g_K_, g_e_ and g_i_. Black dots represent spike onsets (empirical threshold with the first derivative method). B, Predicted threshold vs. membrane potential for the trace in A. Trajectories lie above theoretical threshold on the right of the dashed line (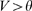). C, Zoom on the second spike in A. Colored lines represent increasingly complex threshold predictions: using Na activation characteristics only (blue, 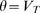), with Na channel inactivation (green, 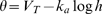), with potassium channel activation (purple, 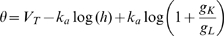) and with synaptic conductances (red, 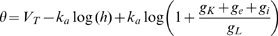). Here the threshold varies mainly after spike onset.

This equation allows us to predict the time-varying value of the threshold from the membrane potential trace (provided that Na inactivation properties are known). The threshold time constant is given by the inactivation time constant (which is voltage-dependent). Fig. 5 shows how the spike threshold varies in a biophysical model with fluctuating synaptic conductances.

The effect of previous spikes on spike threshold, which is presumably due to slow Na inactivation [12], can be understood by looking at how an action potential acts on the inactivation variable h. Typical equilibrium curves for Na inactivation 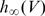 are Boltzmann functions with half-activation values 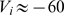
mV and Boltzmann coefficients 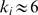 mV [51], so that 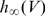 is close to 0 after spike initiation. Thus during the action potential, the inactivation variable relaxes to 0 according to the following equation:
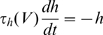
If we note 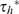 the average value of the time constant 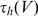 during the action potential and 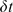 the spike duration (typically, a few ms), then the effect of an action potential on h is a partial reset: 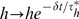, which translates for the threshold into a shift: 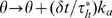. In other words, the spike threshold increases by a fixed amount after each spike, which contributes to the neuron refractory period (see Fig. 5C). This effect was recently demonstrated *in vitro* [22] and explains *in vivo* observations where the threshold was found to be inversely correlated with the previous interspike interval [12]. If the inactivation time constant is long compared to the typical interspike interval, we predict that the threshold should be linearly correlated with the firing rate.

#### Voltage-dependent conductances

In the same way, the dynamics of voltage-dependent conductances translates into threshold dynamics. Potassium currents, in particular Kv1 delayed rectifier currents, are also thought to play a role in threshold modulation [36]–[41]. Let us consider a current with Hodgkin-Huxley-type kinetics: 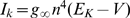, with 
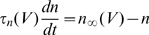
 (n is the activation variable). The corresponding equation for the threshold dynamics then reads (see Materials and Methods):
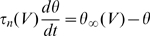
where 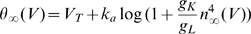 is the equilibrium threshold value (we neglected Na inactivation). Thus, the threshold adapts to the membrane potential. The effect of action potentials can be described similarly as for Na inactivation, except *n* relaxes to 1 during the action potential, yielding the following reset: 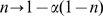. It also results in threshold increase, although it is not additive. This effect also contributes to the neuron refractory period, not only by decreasing the membrane resistance, but also by increasing the spike threshold (see Fig. 5C).

#### Synaptic conductances

Finally, synaptic conductances fluctuate *in vivo*, which also impacts the instantaneous value of the threshold, through the following equation:
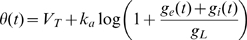
where we neglected Na inactivation and voltage-gated conductances to simplify the formula, and g_e_(t) (resp. g_i_(t)) is the excitatory (resp. inhibitory) synaptic conductance. This formula emphasizes the fact that the threshold equation defines an instantaneous value, which applies to realistic *in vivo* situations where synaptic activity fluctuates. However, we need to make the approximation that fluctuations are slow compared to spike initiation.

Spikes can be triggered either by an increase in the excitatory conductance or by a decrease in inhibitory conductance. In the former case, the total conductance increases and the threshold increases while in the latter case the threshold decreases. In high-conductance regimes (typical of cortical neurons *in vivo*), it has been argued that spikes are mainly triggered by inhibitory decrease because synaptic inhibition is dominant [59], [62]. It might imply that faster depolarization corresponds to lower inhibitory conductance and lower threshold, so that depolarization speed is inversely correlated with spike threshold, as observed experimentally [15]. However, this effect is fundamentally limited by the fact the inhibitory conductance cannot be negative.

### Spike initiation site

#### Effect of neuronal morphology

Spikes are initiated in the axon initial segment (AIS) in spinal motoneurons [63] and in cortical neurons [64], about 35–50 µm from the soma [54], [55]. Our analysis relies on a single-compartment model of spike generation, but the axon hillock is connected with the soma through a large section and with the rest of the axon through a smaller section. To evaluate how electrotonically far the spike initiation site is from the soma, we can compare the length of the AIS to its electrotonic length, given by the following formula [65]:
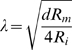
where 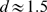
µm is the diameter, 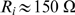.cm is the intracellular resistivity, and 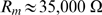.cm^2^ is the membrane specific resistance [66]–[68]. We obtain a value of 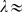
935 µm, many times larger than the distance between the soma and the initiation site. Therefore, below threshold, it is reasonable to consider the soma and AIS as a single electrotonic compartment. Indeed, simultaneous measurements at both sites show that the voltage time course is nearly identical at the two sites before spike initiation [14], [34]. We provide a more detailed analysis in Text S1. We note that the situation changes when an action potential is initiated, because the opening of Na channels reduces the electrotonic length and invalidates the single compartment approximation, which has implications on the shape of action potentials (see Discussion).

For the threshold equation, these considerations imply that conductance values in the equation refer to total conductances over the surface of the soma, proximal dendrites and AIS. Since channel densities are different on these sites, the total conductance for a given ionic channel is 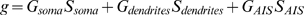, where G_soma_ (resp. G_dendrites_, G_AIS_) is the channel density on the soma (resp. dendrites, AIS) and S_soma_ (resp. S_dendrites_, S_AIS_) is the area of the soma (resp. dendrites, AIS). We give a specific example below.

#### Na channel density in the AIS

Spikes could be initiated in the AIS rather than in the soma because of higher Na channel density [66], [69]–[71], or lower Na half-activation voltage V_a_ [72] in the first segment. Recent experiments and computational modeling suggest that the former hypothesis is more plausible [34], [69], [70]. As an application of our analysis, we can estimate the Na channel density at the AIS using the parameter values reported in [70]. Since Na channels are mainly located in the AIS, we use 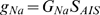. The measured spike threshold (at the AIS) was 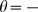
54 mV. To calculate the total leak conductance, we injected a DC current into the soma (using the published model) and obtained g_L_ = 59 nS. We chose this direct method because it is difficult (although possible using linear cable theory) to calculate the total leak conductance using the neuronal morphology, because some of the dendrites may be electrotonically far. The threshold equation relates the threshold value 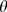 to the values of g_Na_, g_tot_ and the Na channel properties. We can easily invert this relationship, which gives the following formula:
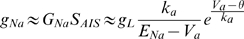
Using the values from Kole et al. (2008) [70] for the channel properties and neuron geometry (V_a_ = −31.1 mV, k_a_ = 6.5 mV, S_AIS_ = 871.3 µm^2^, E_Na_ = 55 mV), we find 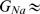
2463 pS/µm^2^, which is very close to the empirically reported value (2500 pS/µm^2^).

### Accuracy of the threshold equation

#### Threshold dynamics in a single-compartment model

**Figure 6:**
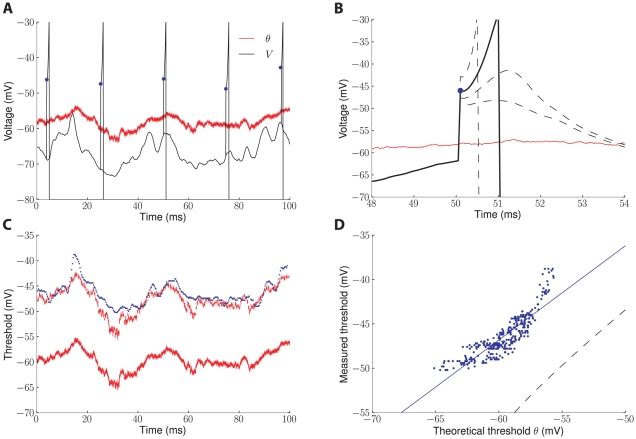
Predicted versus measured dynamical threshold. A, Five superimposed voltage traces of the fluctuating conductance-based model (black traces) stimulated at different times with random depolarization (blue dots show the value of the membrane potential just after the stimulation). Synaptic conductances are identical on all trials. In these examples the stimulations elicited spikes, in other cases (smaller depolarization) they did not. The theoretical threshold is shown in red. B, At a given time (here t = 50 ms), trials with varying depolarization are compared and the measured threshold is defined as the minimal depolarization that elicits a spike (blue dot). C, Predicted threshold (red line) and measured threshold (blue) as a function of time. The shift is mainly due to the fact that the measured threshold is defined with fast inputs (charge threshold) whereas the theoretical threshold is defined with slow inputs: this bias can be calculated and corrected for, as shown by the dashed red line (see also text). D, Measured threshold vs. theoretical threshold for the entire trace (blue dots; blue line: linear regression). The dashed line represents the ideal relationship, taking into account the theoretical difference between threshold for fast inputs and for slow inputs (
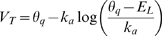
).

To evaluate the quality of the threshold equation, we first simulated a biophysical single-compartment model with fluctuating synaptic conductances, mimicking the effect of synaptic activity in *vivo*. The instantaneous value of the threshold was measured by injecting brief current pulses of varying amplitude in repeated trials with the same synaptic inputs (Fig. 6A, B; see Materials and Methods), and we compared this time-varying value with the prediction from the threshold equation, including the effects of Na inactivation, voltage-gated channels and synaptic conductances. We used this particular stimulation protocol to measure the value of the threshold at any time, rather than only at spike time. We shifted the Na inactivation curve by −12.5 mV so as to obtain more threshold variability (the original model shows little variability). The threshold equation predicted the variations of the measured threshold very well (83% of the variance), with a constant shift which can also be predicted (Fig. 6C, D). This shift has two causes. Firstly, the threshold was measured with brief pulses whereas the predicted threshold corresponds to the definition with slow inputs. Using our formula relating the two definitions (Text S1) indeed reduced this shift from 13.5 mV to 7.4 mV (Fig. 6D). Secondly, because we had to shift the inactivation curve to observe substantial threshold variability, spike threshold was depolarized closer to Na half-activation voltage (−30 mV) and the activation curve is less exponential in that region. Indeed, if V_T_ is calculated as the minimum of the excitability curve rather than with the exponential formula, we find V_T_ = −60.6 mV, which exactly compensates the 7.4 mV shift. When these two predictable biases were taken into account, both the mean and time course of the prediction matched the measured threshold (Fig. 6C, dashed red line). When we did not shift Na inactivation as much, these biases were reduced but the model displayed little variability, which made the prediction less interesting. We address this point in more detail in the Discussion.

**Figure 7 pcbi-1000850-g007:**
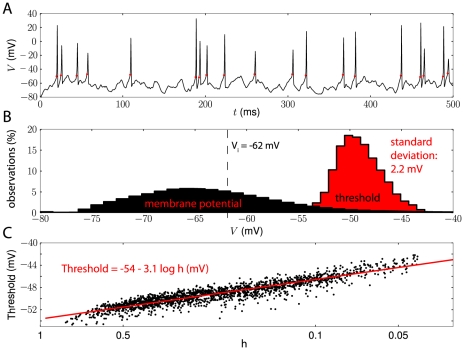
Threshold variability and Na channel inactivation in a single-compartment model. A, We simulated the same model as in Fig. 6, but the half-inactivation voltage V_i_ was shifted to −62 mV (instead of −42 mV in the original model) to increase threshold variability. As a result, spike height was also more variable. B, The threshold distribution (red) spanned a range of more than 10 mV (standard deviation 2.2 mV) and overlapped with the membrane potential distribution (black). C, According to the threshold equation, most threshold variability was due to Na inactivation. Black dots show the measured threshold vs. the inactivation variable *h* (in log scale) at spike times. The linear regression (red line) gives a slope of 3.1 mV, close to the value of k_a_ in this model (Fig. 9D).

In this single-compartment model, threshold variability is much lower than observed *in vivo*. However, the half-inactivation voltage V_i_ in the model is −42 mV, while experimental measurements suggest values around −60 mV in central neurons (e.g. Kole et al. (2008) [70]). According to our analysis, this reduces threshold variability because Na channels do not inactivate below threshold (log h≈0). In Fig. 7, we hyperpolarized V_i_ by 20 mV, giving V_i_ = −62 mV, close to experimental values, and measured the spike threshold with fluctuating inputs (Fig. 7A). We found that the threshold varied over more than 10 mV and the standard deviation 2.2 mV (Fig. 7B), similar to values reported *in vivo* [15]. According to the threshold equation, most threshold variability was due to Na inactivation. A linear regression at spike times gave 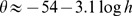 (mV) (Fig. 7C). This 3.1 mV factor is close to the value of k_a_ in this model, as measured by fitting a Boltzmann function to the Na activation curve around −50 mV (see Discussion). We also observe that, in this single-compartment simulation, many spikes were small (Fig. 7A). This is not unexpected, because spikes should be smaller when Na channels are partially inactivated. However, this property should not be taken as a prediction, because it is known that the correct spike shape of cortical neurons cannot be recovered in single-compartment models [10], [13].

#### Threshold prediction in a realistic multicompartmental model of spike initiation

**Figure 8:**
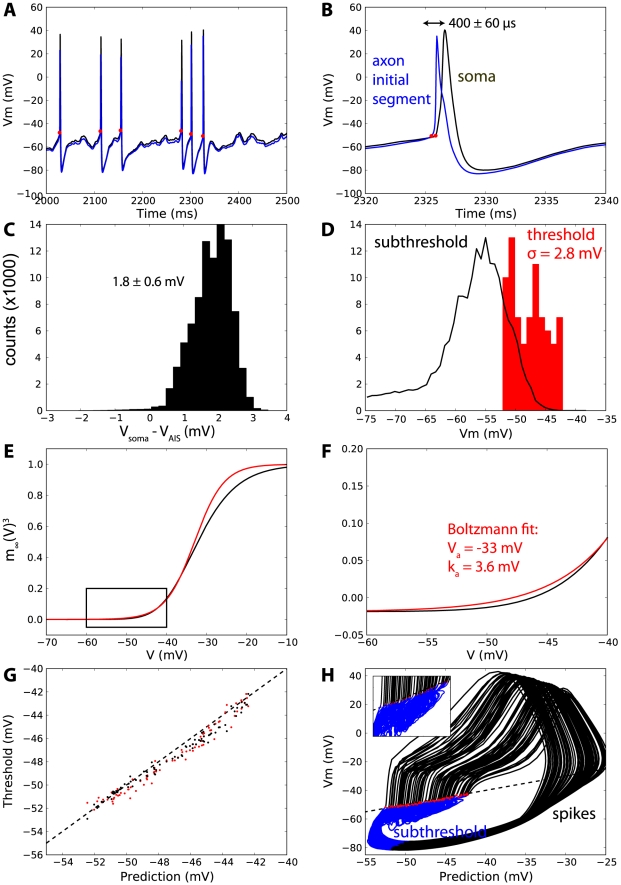
Accuracy of the threshold equation in a multicompartmental model of spike initiation [**54**]. A, Voltage trace at the soma (black) and at the spike initiation site in the axon initial segment (AIS, blue) in response to a fluctuating current. The spike threshold was measured at the soma when dV/dt exceeded 10 V.s^−1^ (red dots). B. Zoom on an action potential: spikes were initiated at the AIS 400±60 µs before observed at soma. C. Between spikes, the membrane potential was slightly higher at the soma than at the AIS (1.8±0.6 mV). D. The spike threshold (measured at the soma) was very variable (standard deviation 2.8 mV): its distribution spanned 10 mV (−52 to −42 mV) and significantly overlapped the subthreshold distribution of the membrane potential (i.e., with spikes removed). E, F. We fitted the activation curve of the Nav1.6 channel (black) to a Boltzmann function (red) in the spike initiation zone (rectangle and panel F), yielding V_a_ = −33 mV and k_a_ = 3.6 mV. G. Measured threshold (red: at the soma, black: at the AIS) vs. theoretical prediction for all spikes. The dashed line represents equality (measurement = prediction). H. Somatic membrane potential vs. theoretical threshold at all times. Spikes are shown in black (defined as voltage trace 7 ms from spike onset), subthreshold trajectories in blue and spike times as red dots: spikes are indeed initiated when the membrane potential exceeds the theoretical threshold (inset: zoom on spike onsets).

We then checked the accuracy of the threshold equation with a realistic multicompartmental model of spike initiation, where action potentials are initiated in the axon [54]. We injected a fluctuating current in the soma and compared measured spike thresholds with our theoretical predictions (Fig. 8). Spikes were initiated in the axon 400±60 µs earlier than observed at the soma (Fig. 8B). When action potentials were removed from the voltage traces, the membrane potential was 1.8±0.6 mV higher at the soma than at the spike initiation site in the axon initial segment (AIS; Fig. 8C). The threshold measured at the soma was −47.7±2.8 mV and varied between −52.1 mV and −42.2 mV (Fig. 8D). Its distribution significantly overlapped the subthreshold distribution of the membrane potential, as observed *in vivo*. We estimated the activation properties of the Nav1.6 channel, which is responsible for spike initiation in this model, by fitting a Boltzmann function to the activation curve (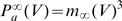) in the spike initiation zone (−60 mV to −40 mV). We found V_a_ = −33 mV and k_a_ = 3.6 mV (Fig. 8E, F). This is different from experimentally reported values (in particular, k_a_ is smaller) because these were obtained by fitting the activation curve on the entire voltage range. We address this specific point in the Discussion. We then calculated the total maximal conductance of the Nav1.6 channels (over the AIS), the slow K+ channels (Km) and the fast K+ channels (Kv), using the published morphology and channel density (see Materials and Methods).

Using these estimated values and the time-varying values of the channel variables (h, n_Km_, n_Kv_^4^) at the AIS, we calculated the theoretical threshold at all times, and compared the prediction with the measured threshold at spike times (Fig. 8G). Values of the channel variables were taken at the time of spike initiation in the AIS and the threshold was measured at the AIS (black) and at the soma (red). The prediction with the threshold equation was very good: the average error was 0.7 mV. The threshold prediction was on average only 0.49 mV higher than the measured threshold. However, this excellent match is probably fortunate because the value of the measured threshold is correlated with the measurement criterion (on dV/dt) and in general, we would expect a constant shift between prediction and measurement. When this shift was removed, the average prediction error was 0.53 mV. Among the different contributions to the threshold, we found that only Na inactivation had a significant impact. The fast K+ current (IKv) had a very high maximum conductance but was only activated after spike initiation, while the slow K+ current (IKm) had a small maximum conductance. According to the threshold equation, total conductance contributed only 0.07 mV to threshold variability. A linear regression gave 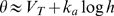 with V_T_ = −56mV and 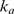 = 3.6 mV, very close to our predicted values, and the average estimation error with this formula was 0.08 mV.

These results show that the value of the membrane potential at spike onset is well predicted by the threshold equation. However, to prove that our equation really defines a spike threshold, we also need to show that the membrane potential is always below the predicted threshold before spikes. In Fig. 8H, we plotted the membrane potential vs. the predicted threshold for the entire voltage trace (5 seconds). It clearly appears that the neuron spikes when its membrane potential exceeds the predicted threshold, and that the potential is always below threshold between spikes.

## Discussion

The spike threshold differs between cells and for different types of stimulations [2], [15], [32], [33], [35]. We have identified several modulation factors, whose quantitative influence is summarized by the *threshold equation*:
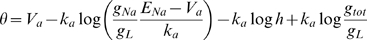
That formula relates the value of the threshold to the activation and inactivation properties of the Na channel, the properties of other voltage-gated channels such as Kv1 and synaptic conductances (g_tot_ is total conductance, excluding Na conductance). It consists of a static part (first two terms), determined by the properties of Na channel activation, and of a dynamic part, which depends on the proportion of inactivated Na channels (1-h) and on the total conductance of other channels.

It describes the voltage threshold at the site of spike initiation (rather than at the soma), and is correlated but not identical to empirical “threshold” measures, which measure spike onset rather than threshold (those normally overestimate the threshold). From that formula, we were able to derive a dynamical equation for the instantaneous threshold, which explains the variability of the spike threshold in the same cell and predicts its relationship with previous membrane potential history. We found that the threshold equation was a good predictor of the time-varying threshold in biophysical models with fluctuating inputs (Fig. 6–8).

### Mechanisms for threshold modulation and variability

Since Na channels are responsible for the generation of action potentials, the threshold is firstly determined by their activation characteristics. Activation curves for Na channels are well approximated by Boltzmann functions with similar slope factors (k_a_ = 4–8 mV in neuronal channels). The threshold is linearly related to the half-activation value V_a_ and logarithmically related to the maximum Na conductance g_a_. The threshold also depends logarithmically on the Na inactivation variable h, so that it increases with the membrane potential and with every emitted spike. The modulating effect of inactivation is most pronounced when the half-activation value V_i_ is lowest (i.e., Na channels are partially inactivated at rest). Finally, the threshold depends logarithmically on the total conductance, which includes the leak conductance, voltage-gated conductances and synaptic conductances. In particular, Kv1 channels, which are expressed with high density at the spike initiation site [37], [39], [41], increase the threshold in an adaptive manner (the threshold increases with the membrane potential). This change in threshold occurs simultaneously with the effective membrane time constant, whereas threshold changes due to Na inactivation have no effect on the time constant, which might suggest a way to experimentally distinguish between the two effects. Indeed, the effective membrane time constant (as measured *in vivo* for example in Léger et al., 2003 [73]) is 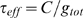 (C is the membrane capacitance) while the effect of total conductance on spike threshold varies as 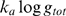, therefore as −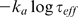. It is currently unclear whether threshold modulation is mainly due to Na inactivation or delayed-rectifier K currents. Our simulations with the multicompartmental model of spike initiation in pyramidal cells [54] suggest that the spike threshold is essentially determined by Na inactivation, but this may not be universally true. Recent experimental findings in hippocampal mossy fibers [74] suggest that delayed K+ currents are closed at spike initiation, which minimizes charge movements across the membrane and is thus more metabolically efficient. It emphasizes the fact that Na inactivation is a more metabolically efficient way to modulate spike threshold than K+ activation, since the former reduces charge transfer while the latter increases it.

We have not considered the effect of channel noise, i.e., fluctuations in Na channel gating [42], [75]–[78], which result in random threshold variations. Although dynamical equations of fluctuations in Na channel gating are well set [79], [80], they cannot be included in our theoretical framework because we neglected the time constant of Na activation (which leads to the exponential model).

There are two additional sources of variability which are artefactual: the fact that the threshold is not measured at the site of spike initiation, and threshold measurement methods. The latter source is difficult to avoid *in vivo* because only spike onsets can be measured. The former one also seems technically very difficult to avoid *in vivo*, since spikes are initiated in the axon hillock, which is only a few microns large. Although the soma and AIS are virtually isopotential below threshold, experimentally measured values of threshold differ between the two sites [34] because, as we previously remarked, *in vivo* measurements correspond to spike onset rather than threshold and therefore take place after spike initiation, when the two sites are not isopotential anymore. This experimental difficulty may introduce artefactual variability in threshold measurements [14].

### Approximations in the threshold equation

To derive the threshold equation, we made several simplifying assumptions. First, we assumed that Na activation is instantaneous. It is indeed significantly faster than all other time constants but not instantaneous. The approximation is legitimate as long as the effective membrane time constant in the membrane equation is small (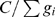, including all conductances), which is generally true before threshold. When Na channels open, the Na conductance dominates the total conductance and drastically reduces the effective time constant. Thus, we expect this approximation to be reasonable to predict spike initiation properties but not spike shape characteristics. Our second major assumption is a quasistatic approximation, i.e., we assume that near spike initiation, all modulating variables and the input current can be considered as constant. In other words, we assume that the time constants (except that of Na activation) are larger than a few ms. This is clearly only a mathematically convenient approximation, but our predictions empirically agreed with numerical simulations. To investigate the role of Na inactivation, we also assumed that activation and inactivation are independent, which is a standard simplifying hypothesis (Hille, 2001). Although it is debatable [49], [56], it should remain valid in the case where activation and inactivation time constants are well separated.

**Figure 9:**
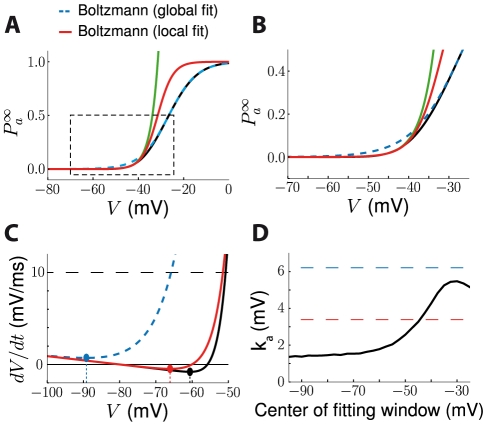
Fitting the Na activation curve to a Boltzmann function. A, The Na channel activation curve of the conductance-based model (black line) was fit to a Boltzmann function on the entire voltage range (dashed blue line) and on the spike initiation range only (−60 mV to −40 mV, red line). The green line shows the exponential fit on the spike initiation range. B, In the hyperpolarized region (zoom of the dashed rectangle in A), the global Boltzmann fit (dashed blue line) is not accurate, while the local Boltzmann fit and the local exponential fit better match the original curve. C, For hyperpolarized voltages (<−50 mV), the resulting excitability curve is closer to the original curve (black) with a local Boltzmann fit (red) than with a global fit (dashed blue), yielding more accurate threshold estimations (dots). D, The estimated Boltzmann slope k_a_ is very sensitive to the position of the fitting window and varies between 2 mV and 6 mV.

**Figure 10:**
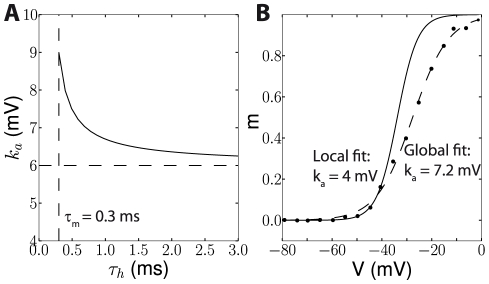
Experimental difficulties in the measurement of Na activation curves. A, Estimation of the activation slope k_a_ from simulated voltage-clamp data as a function of the inactivation time constant τ_h_. The model was of a membrane with only Na channels, and activation and inactivation curves were Boltzmann functions (see Materials and Methods). The activation slope was measured by a Boltzmann fit in the hyperpolarized region (<−40 mV). The activation slope k_a_ was 6 mV in the model (dashed line), but the measurement overestimated it when the inactivation time constant was very close to the activation time constant. B, Na activation curve measured in vitro (dots, digitized from [86]) and Boltzmann fits over the entire voltage range (dashed curve) and over the hyperpolarized range (V<−40 mV, solid curve).

We also assumed that Na activation and inactivation curves were Boltzmann functions. Experimental data is indeed well fitted by Boltzmann functions, but the reported parameter values (V_a_, k_a_) correspond to fits on the entire voltage range, whereas we are interested in hyperpolarized voltage regions where the activation values are small. When only the relevant part of the experimental data is considered, different parameter values might be obtained. For example, when analyzing previously published biophysical models, we found that better results were obtained when Na activation curves, which were not exactly Boltzmann functions, were fitted in the spike initiation region (−60 to −40 mV) rather than on the entire voltage range (Fig. 9). We examined this issue in the biophysical model used in this paper (see Materials and Methods). The Na activation curve of this model seemed to be well fit to a Boltzmann function (Fig. 9A), however the fit was poor in the spike initiation zone (−60 to −40 mV, Fig. 9C) where activation is close to zero, which makes fit errors relatively larger. Although the slope factor 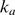 is about 6 mV when the activation curve is fit over the entire voltage range, similar to experimental measurements [51], it is only half this value when fit in the spike initiation region (Fig. 9D), which explains why this model, as many other biophysical models, exhibits little threshold variability (since threshold modulation is proportional to 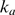). We calculated the slope factor as a function of the voltage region and we found that it varies between 1 and 6 mV (Fig. 9D). This finding motivates a reexamination of Na channel voltage-clamp data, focusing on the spike initiation region rather than on more depolarized regions, which are more relevant for spike shape than spike initiation. Fig. 10 addresses two potential difficulties. In experiments, activation curves are obtained by measuring the peak conductance after the clamp voltage is changed from an initial value V_0_ to a target value V, and normalizing over the entire range of target voltages. Thus, it assumes that inactivation is still at resting level h(V_0_) when the peak current is measured. This would not be the case if the inactivation time constant τ_h_ were close to the activation time constant τ_m_. Fig. 10A shows the effect of this overlap on the measurement of k_a_ with simulated voltage-clamp data, where 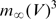 is a Boltzmann function with k_a_ = 6 mV. It appears that k_a_ is overestimated if τ_h_ is very close to τ_m_, up to 50% when the two time constants are equal (to 0.3 ms in these simulations). However the error quickly decreases as τ_h_ increases (e.g. 10% error for τ_h_ = 1 ms). Another potential difficulty is the lack of data points in the relevant voltage range and the measurement noise, because currents are small. In Fig. 10B, we digitized an experimentally measured activation curve (black dots), where clamp voltages were spaced by 5 mV. A Boltzmann fit over the entire voltage range gave k_a_ = 7.2 mV while a fit over the hyperpolarized region V<−40 mV gave k_a_ = 4 mV. However, the latter is not a reliable estimate because it corresponds to only 4 non-zero data points, which also seem to be corrupted by noise. Therefore it might be necessary to perform new measurements, specifically focusing on the spike initiation zone, perhaps with multiple measurements to reduce the measurement noise. Alternatively, k_a_ could be measured with a phenomenological approach, using white noise injection in current clamp [22]. Another possible approach would be to directly fit the excitability model to the current-clamp response of a cell in which only Na channels would be expressed (perhaps with fluctuating inputs).

Finally, our analysis relies on a single compartment model. In the compartmental model, we found that between spikes, the membrane potential was 1.8±0.6 mV more depolarized at the soma than at the AIS. This is small compared to the slopes of all activation and inactivation curves in this model (5–9 mV). This agrees with our analysis of the electrotonic length in the subthreshold range, which is much larger than the distance between the soma and the AIS, although very fast synaptic inputs or proximal axonal inhibition could produce larger voltage gradients. Thus, our analysis should remain valid if the compartment represents both the soma and initiation site (and also proximal dendrites). However, that approximation is not valid anymore after spike initiation (see below).

### Sharpness of spikes and threshold variability

Spikes look sharper in the soma than in the AIS, presumably because they are initiated in the AIS and back-propagated to the soma [10], [13], [14]. That property is also seen in numerical simulations of multicompartmental models [34], [70]. Yet, linear cable theory predicts the opposite property: the voltage at the soma is a low-pass filtered version of the voltage at the AIS, therefore spikes should look less sharp in the soma. Thus, increased sharpness must be due to active backpropagation of the action potential, which cannot be seen in a two compartment model (such as described in Text S1). From a theoretical point of view, the sharpening effect of backpropagation can be intuitively understood from the cable equation:
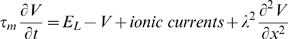
It appears that the membrane equation is augmented by a diffusion term, which is positive and large in the rising phase of the action potential between the initiation site and the soma. Thus, for the same membrane potential V, the time derivative gets larger as this diffusion term increases, which sharpens action potentials.

Sharpness can be measured in numerical simulations by plotting dV/dt vs. V in response to a suprathreshold DC current, and fitting it to an exponential model (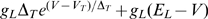). In the model of Hu et al. [54], we found that the slope factor, characterizing spike sharpness, was 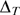 = 1.6 mV in the AIS and only 0.8 mV in the soma. This is in approximate agreement with empirical fits of exponential integrate-and-fire models to cortical neurons stimulated with fluctuating inputs, which reveal a surprisingly small slope factor 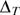, slightly above 1 mV [22]. Thus, in the multicompartmental model, active backpropagation did increase spike sharpness in the soma, but also in the AIS, since the slope factor was about twice smaller than predicted from fitting the Na activation curve to a Boltzmann function (3.6 mV). This increased sharpness did not affect the magnitude of threshold modulation. In single-compartment models, sharpness of spikes and threshold modulation are determined by the same quantity, related to the sharpness of the Na activation curve (k_a_). It appears that this link does not hold anymore when active backpropagation is considered (in multicompartmental models). Thus, in the threshold equation, the modulating factor is indeed k_a_ (from the Na activation curve) rather than 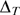 (from spike sharpness, measured in the phase plot (dV/dt, V)). This explains that Na inactivation can produce large threshold variability (10 mV in our simulations) even though spikes are very sharp.

## Materials and Methods

### Membrane equation

We consider a single-compartment neuron model with voltage-gated sodium channels and other ion channels (voltage-gated or synaptic), driven by a current I. The membrane potential V is governed by the membrane equation:
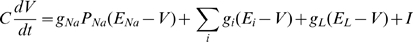
where C is the membrane capacitance, g_L_ (resp. E_L_) is the leak conductance (resp. reversal potential), g_i_ (resp. E_i_) is the conductance (resp. reversal potential) of channel i, g_Na_ (resp. E_Na_) the maximum conductance (resp. reversal potential) of sodium channels, P_Na_ is the proportion of open Na channels and I is the input current. In this article, we used the following convention for conductances: lower case (g) for the total conductance over the surface of a compartment (typically in units of nS) and upper case (G) for conductances per unit area (in units of S/cm^2^).

We assume that sodium channel activation and inactivation are independent, as in the Hodgkin-Huxley model [3], i.e., 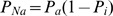, where P_a_ is the probability that activation gates are open and P_i_ is the probability that a channel is inactivated. Following the Hodgkin-Huxley formalism, we define 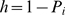. The steady-state activation curve 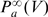 can be empirically described as a Boltzmann function [51]:
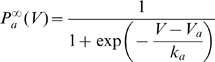
where 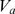 is the half-activation voltage (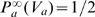) and 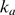 the activation slope factor (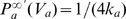). We make the approximation that Na activation is instantaneous and we replace P_a_ by its equilibrium value, so that 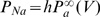.

### Exponential approximation

With instantaneous activation, the sodium current is:
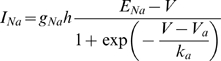
Action potentials are initiated well below V_a_ (about −30 mV, Angelino and Brenner, 2007 [51]), so that 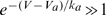 except during the spike. Similarly, E_Na_ is very high (about 55 mV), so that 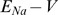 is not very variable below threshold. We make the approximation 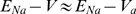 and we obtain:
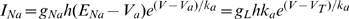
where 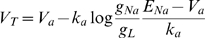. This approximation is meaningful for spike initiation but not for spike shape. With a reset (ignoring inactivation and other ionic channels), we obtain the exponential integrate-and-fire model [48], which predicts the response of cortical neurons to somatic injection with good accuracy, in terms of spike timings [22], [81], [82]. In this model, V_T_ is the voltage threshold for constant input currents I and k_a_ (originally denoted Δ_T_) is the slope factor, which measures the sharpness of spikes: in the limit 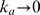 mV, the model becomes a standard integrate-and-fire model with threshold V_T_ (although this is different in multicompartmental models, see Discussion). The resulting approximated model is thus:
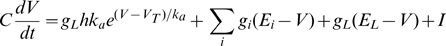
It is convenient to sum all conductances (except for the Na channel), which gives a simpler expression:
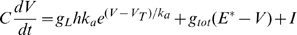
where 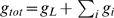 is the total conductance and E* is the effective reversal potential:
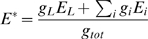
Finally, the inactivation variable h can be inserted in the exponential function:
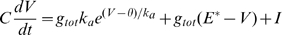
where
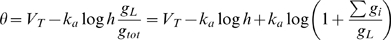
is the voltage threshold if all other variables are constant, i.e., it is such that F′(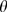) = 0, where F is the current-voltage function.

### Dynamic threshold

The effect of Na inactivation on the threshold can be seen in the exponential model above, neglecting other conductances (thus 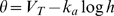). Assuming that inactivation is slow compared to spike initiation (quasi-static approximation), the voltage threshold is now 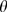, and it changes with the inactivation variable h. We assume, as in the Hodgkin-Huxley model, that inactivation has first-order kinetics:
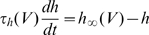
The steady-state value of the threshold is thus 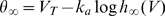. We differentiate the threshold equation with respect to time:
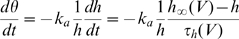
We now express *h* as a function of 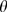 using the invert relationships: 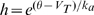 and 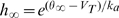:
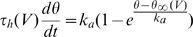
If the threshold remains close to its steady-state value (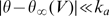), the equation simplifies to:
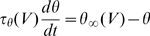
with 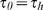. The same method applies for voltage-gated conductances (e.g. Kv1).

### Numerical simulations

We compared our theoretical predictions with numerical simulations of a previously published point-conductance model with fluctuating synaptic inputs [83]. The membrane equation is:
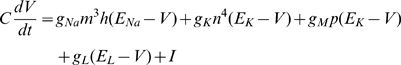
where 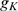 and n are respectively the maximal conductance and the activation variable of thedelayed-rectifier potassium current, and 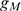 and p are respectively the maximal conductance and the activation variable of the non-inactivating K current. All channel variables have standard Hodgkin-Huxley type dynamics.

In Fig. 3A–C, only Na channel activation was considered, with instantaneous dynamics, i.e., 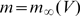, h = 1, n = 0, p = 0, I = 0:
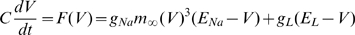
In Fig. 3D, the threshold equation was used to calculate V_T_ for the Na channel properties reported in Angelino and Brenner (2007) [51], since only the values of V_a_ and k_a_ were available.

To evaluate our threshold equation with time-varying inputs (Figs. 2C, 5 and 6), we simulated the full conductance-based model with fluctuating synaptic conductances (same parameters as in Destexhe et al., 2001 [83]). In Fig. 6, we shifted the voltage dependence of Na inactivation toward hyperpolarized potentials by −12.5 mV so as to obtain more threshold variability. To measure the time-varying threshold, we used a similar method as one previously used *in vitro* by Reyes and Fetz [84], [61]. We simulated the model for 200 ms and measured the instantaneous threshold 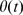 at regular time intervals T as follows. The model was simulated repeatedly with the same synaptic inputs (frozen noise). In each trial, the neuron was depolarized at time nT (only once per 100 ms run) to a voltage value between −51 mV and −38 mV. With T = 0.6 ms and 65 voltage values, we ran 22,000 trials. The threshold at a given time is defined as the minimal voltage value above which a spike is elicited. The measured threshold was compared to the prediction obtained with the threshold equation (see Results), where V_T_ and *k_a_* were obtained from a Boltzmann fit to the activation function 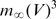 over the range −51 mV to −38 mV, giving V_T_ = −68 mV and k_a_ = 3.7 mV (V_a_ = −30.4 mV). The values of V_T_ and k_a_ depended on the fitting window (see Discussion and Fig. 9). In Fig. 7, the voltage dependence of Na inactivation was shifted by −20 mV to induce more threshold variability (giving V_i_ = −62 mV instead of −42 mV with the original parameter values) and the maximum Na conductance was multiplied by 3 (to keep threshold values in the same range). The standard deviations of synaptic conductances were also increased.

In Fig. 8, we simulated a multicompartmental model of spike initiation recently published by Hu et al. (2009) [54], with fluctuating injected current modeled as an Ornstein-Uhlenbeck process (mean 0.7 nA, standard deviation 0.2 nA, time constant 10 ms). The model was otherwise unchanged. The spike threshold, both at the soma and AIS, is defined at the voltage value when dV/dt first exceeds 10 V.s^−1^ preceding a spike. In some panels (Fig. 8C, D, H), we extracted spikes from voltage traces by removing parts between spike onsets and 7 ms later. We estimated the activation properties of the Nav1.6 channel, which is responsible for spike initiation in this model, by fitting a Boltzmann function to the activation curve (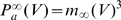) in the spike initiation zone (−60 mV to −40 mV), which gave V_a_ = −33 mV and k_a_ = 3.6 mV. We then calculated the total maximal conductance of the Nav1.6 channel over the AIS, by integrating the channel density over the surface of the AIS (using the morphology and channel density implemented in the published model code). We found g_Na_ = 236 nS. Calculating the total leak conductance in this way was more difficult because leak channels were uniformly distributed on the whole morphology, including the dendrites, so that spatial attenuation should be taken into account. While this is theoretically possible using linear cable theory, we chose a simpler approach by directly measuring the membrane resistance at the soma with a DC current injection, and we found g_L_ = 38 nS. With these values, the threshold equation predicted that the base threshold is V_T_ = −55.9 mV. The model had a slow K+ current (Im) with the same channel density as the leak channels. Therefore the maximum total conductance was estimated as g_Km_ = g_L_ = 38 nS. It also had a fast K+ current which was distributed inhomogeneously on the whole neuron morphology, including dendrites. We estimated its total maximum conductance as 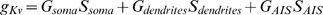, where the effective dendritic area was estimated from the ratio of total leak conductance over leak channel density, i.e., 
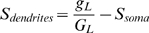
. We found g_Kv_ = 906 nS. We then calculated the theoretical threshold using these parameters and the instantaneous values of the relevant channel variables (h, n_Km_, n_Kv_^4^).

In Fig. 10A, we simulated a voltage clamp experiment in a simplified model with only Na channels, assuming the leak current was subtracted, where both activation and inactivation curves (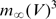 and 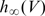) were Boltzmann functions, with parameters V_a_ = −30 mV, k_a_ = 6 mV, V_i_ = −65 mV and k_i_ = 6 mV. The activation and inactivation time constant were fixed (τ_m_ = 0.3 ms and τ_h_ between 0.3 and 3 ms). The conductance was measured at the current peak after the clamp voltage was switched from a fixed initial voltage V_0_ = −70 mV to a test voltage V, which was varied between −100 and 50 mV (the current was divided by V-E_Na_ to obtain the conductance, and we assumed that E_Na_ was known - in an experiment it would be obtained from a linear fit to the highest voltage region). The conductance was normalized by the maximal conductance over the tested voltage range and the resulting curve was fit to a Boltzmann function in the hyperpolarized region V<−40 mV.

All simulations were written with the Brian simulator [85] on a standard desktop PC, except the simulation of the multicompartmental model of Hu et al. [54], for which we used Neuron.

## Supporting Information

Text S1 Supplementary Methods. A) Relationship between threshold definitions. B) Two compartments model.
(0.09 MB PDF)
