# Supplementary material for: A Threshold Equation for Action Potential Initiation
Source: PLoS Comput Biol. 2010 Jul 8;6(7):e1000850. doi: 10.1371/journal.pcbi.1000850 (PMC2900290; doi:10.1371/journal.pcbi.1000850)
Supplement: Text S1 — Supplementary Methods. A) Relationship between threshold definitions. B) Two compartments model. (0.09 MB PDF) [file pcbi.1000850.s001.pdf]

A Threshold Equation for Action Potential Initiation

Jonathan Platkiewicz[1,2] and Romain Brette[1,2] (romain.brette@ens.fr)
1. Laboratoire Psychologie de la Perception, CNRS and Université Paris Descartes, Paris, France
2. Département d'Etudes Cognitives, Ecole Normale Supérieure, Paris, France

Text S1 - Supplementary Methods

A. Relationship between threshold definitions

To examine the relationship between threshold definitions, we consider the exponential approximation of the membrane equation:

$$C\frac{dV}{dt} = F(V) + I = g_L(E_L - V) + g_L\Delta_T e^{\frac{V-V_T}{\Delta_T}} + I$$

where V_T is the voltage threshold for slow inputs. The threshold θ_q for fast inputs is defined with very short current pulses $I(t) = q\delta(t)$ (where q is the total charge) as the voltage value for the threshold charge q_{th}, which is the larger voltage such that $F(\theta_q)=0$:

$$\frac{\theta_q - E_L}{\Delta_T} = \exp\left(\frac{\theta_q - V_T}{\Delta_T}\right)$$

which simplifies to:

$$\theta_q = V_T + \Delta_T\log\left(\frac{\theta_q - E_L}{\Delta_T}\right)$$

This relationship gives V_T as a function of θ_q. To invert this formula, we may observe that the second term is a small correction to the threshold V_T, which suggests the following approximation:

$$\theta_q \approx V_T + \Delta_T\log\left(\frac{V_T - E_L}{\Delta_T}\right)$$

The empirical threshold measure θ_e defined by the first derivative method ($dV/dt=k_{th}$) can be related to V_T in the same way:

$$\theta_e \approx V_T + \Delta_T\log\left(\frac{V_T - (E_L + RI - \tau k_{th})}{\Delta_T}\right)$$

where $\tau = C/g_L$ is the membrane time constant ($R=1/g_L$ is the membrane resistance). Importantly, the corrective term depends on the total conductance, in a way that tends to increase the modulating effect of conductances on the measured threshold.

B. Two compartments model

Here we consider a model with two compartments, representing the soma and the AIS. It is described by two equations:

$$C_{AIS}\frac{dV_{AIS}}{dt} = g_{Na}^{AIS}P_a^{\infty}(V_{AIS})(E_{Na} - V_{AIS}) + g_L^{AIS}(E_L - V_{AIS}) + g_c(V_{soma} - V_{AIS})$$

$$C_{soma}\frac{dV_{soma}}{dt} = g_L^{soma}(E_L - V_{soma}) + g_c(V_{AIS} - V_{soma}) + I$$

where we have neglected the Na current in the soma, because it does not play a role in spike initiation (only in the second phase of the action potential), and g_c is the coupling conductance between the two compartments. The threshold value at the AIS for slow depolarizations can be calculated similarly as in the single-compartment model, i.e., the voltage at the bifurcation point.

When I is slowly increased, both voltage derivatives are zero, so that the two equations can be written as:

$$0 = g_{Na}^{AIS} P_a^{\infty}(V_{AIS})(E_{Na} - V_{AIS}) + g_L^{AIS}(E_L - V_{AIS}) + g_L^{soma}(E_L - V_{soma}) + I$$

$$0 = g_L^{soma}(E_L - V_{soma}) + g_c(V_{AIS} - V_{soma}) + I$$

It appears that V_{soma} can be expressed as a linear function of V_{AIS}, so that the first equation becomes

$$0 = g_{Na}^{AIS} P_a^{\infty}(V_{AIS})(E_{Na} - V_{AIS}) + g_L{}^*(E_L^* - V_{soma}) + I^*$$

where $g_L^* = g_L^{AIS} + g_L^{soma} \frac{g_c}{g_L^{soma} + g_c}$ and I^* and E_L^* are constants (their value is irrelevant here). This equation is formally identical to the one obtained from a single compartment model with conductance g_L^*, which we call the *effective* leak conductance, so the threshold equation is the identical, except g_L must replaced by g_L^*. That result can be understood intuitively as follows: if spike initiation is electrotonically far from the soma, then the coupling conductance is small and g_L^* is the leak conductance near the initiation site; if spike initiation is near the soma (which seems to be the case), then the soma and AIS are close to isopotential, so that g_L is the total conductance over the two compartments. Experimental studies have shown that spikes are initiated about 50 µm from the soma [1, 2]. The coupling conductance is $g_c = \frac{\pi d^2}{4 R_i l}$, where $d \approx 1 - 2$ µm is the average diameter of the AIS, $l \approx 35 - 50$ µm is its length and $R_i \approx 100$ Ω.cm is the intracellular resistivity, yielding $g_c \approx 30 - 90$ nS, which is very high. Indeed, assuming the somatic surface is about 1500 µm^2 [3] and $G_L \approx 0.1$ mS/cm^2, the total somatic leak conductance is only $g_L^{soma} \approx 1.5$ nS. Therefore, for the threshold calculation, we can assume that the soma and AIS are isopotential. However, the conductances are not homogeneously distributed on both sites, so that all conductances should be calculated as $g = G_{soma} S_{soma} + G_{AIS} S_{AIS}$ (S_{soma} and S_{AIS} are the somatic and AIS areas, respectively), as explained in Results.

Forward and backward propagation

The two-compartments model may be used to calculate the propagation delay between the soma and initiation site. Because of the geometrical asymmetry, the forward and backward delays are different.

Firstly, below threshold, the difference $u = V_{AIS} - V_{soma}$ follows a linear differential equation:

$$\tau_u \frac{du}{dt} = -u - \frac{\tau_u}{C S_{soma}} I$$

where

$$\tau_u = \frac{C}{G_L + g_c \left(\frac{1}{S_{AIS}} - \frac{1}{S_{soma}} \right)} \approx \frac{C S_{AIS}}{g_c}$$

is the time constant, and C is the specific membrane capacitance (about 0.9 µF/cm^2, Gentet et al., 2000 [4]). Using the values above, we find $\tau_u \approx 25 - 100$ µs, meaning that the voltage at the AIS follows that at the soma with a very short delay (below threshold). When a spike is initiated in the AIS and backpropagated, the characteristic time constant of the somatic membrane equation gives us the backpropagation delay (which is an integration delay):

$$\tau_{spike} = \frac{C_{soma}}{g_L^{soma} + g_c} \approx \frac{C S_{soma}}{g_c}$$

With the same parameter values as previously, we obtain $\tau_{spike} \approx 100 - 900$ µs. Palmer and Stuart (2006) reported $\tau_{spike} = 150$ µs in layer 5 pyramidal neurons [5]. An interesting point to

note is that passive forward propagation is significantly faster than action potential backpropagation, as observed experimentally, simply because the axon is smaller than the soma.

References

1. Hu W, Tian C, Li T, Yang M, Hou H, et al. (2009) Distinct contributions of Na(v)1.6 and Na(v)1.2 in action potential initiation and backpropagation. Nat Neurosci 12: 996–1002.

2. Kress GJ, Mennerick S (2009) Action potential initiation and propagation: upstream influences on neurotransmission. Neuroscience 158: 211–222.

3. Mainen ZF, Joerges J, Huguenard JR, Sejnowski TJ (1995) A model of spike initiation in neocortical pyramidal neurons. Neuron 15: 1427–1439.

4. Gentet LJ, Stuart GJ, Clements JD (2000) Direct measurement of specific membrane capacitance in neurons. Biophys J 79: 314–320.

5. Palmer LM, Stuart GJ (2006) Site of action potential initiation in layer 5 pyramidal neurons. J Neurosci 26: 1854–1863.

6. Aizenman CD, Akerman CJ, Jensen KR, Cline HT (2003) Visually driven regulation of intrinsic neuronal excitability improves stimulus detection in vivo. Neuron 39: 831–842.
